# Serum cytokine levels and other associated factors as possible immunotherapeutic targets and prognostic indicators for lung cancer

**DOI:** 10.3389/fonc.2023.1064616

**Published:** 2023-02-16

**Authors:** Yinghao Zhao, Shengnan Jia, Kun Zhang, Lian Zhang

**Affiliations:** ^1^ Department of Thoracic Surgery, The Second Hospital of Jilin University, Changchun, China; ^2^ Department of Hepatopancreatobiliary Medicine, The Second Hospital of Jilin University, Changchun, Jilin, China; ^3^ Department of Central Lab, The Second Hospital of Jilin University, Changchun, China; ^4^ Department of Pathology, The Second Hospital of Jilin University, Changchun, China

**Keywords:** serum cytokines, targeted immunotherapy, prognosis, biomarkers, lung cancer

## Abstract

Lung cancer is one of the most prevalent cancer types and the leading cause of cancer-related deaths worldwide. Non-small cell lung cancer (NSCLC) accounts for 80-85% of all cancer incidences. Lung cancer therapy and prognosis largely depend on the disease’s degree at the diagnosis time. Cytokines are soluble polypeptides that contribute to cell-to-cell communication, acting paracrine or autocrine on neighboring or distant cells. Cytokines are essential for developing neoplastic growth, but they are also known to operate as biological inducers following cancer therapy. Early indications are that inflammatory cytokines such as IL-6 and IL-8 play a predictive role in lung cancer. Nevertheless, the biological significance of cytokine levels in lung cancer has not yet been investigated. This review aimed to assess the existing literature on serum cytokine levels and additional factors as potential immunotherapeutic targets and lung cancer prognostic indicators. Changes in serum cytokine levels have been identified as immunological biomarkers for lung cancer and predict the effectiveness of targeted immunotherapy.

## 1 Introduction

Lung cancer or lung carcinoma is the most common cause of cancer incidence and cancer-related deaths worldwide (1). Annually, over 2.2 million people are affected, and it causes around 1.8 million deaths worldwide. It has the highest incidence in developing nations, where tobacco use is most prevalent (2). Lung cancer mortality is projected to have grown by 18.3% between 2006 and 2016; in highly developed nations, attributable years of life lost came in second. New statistics show that in 2019, lung cancer was the seventeenth largest cause of disability-adjusted life years (DALYs) for all ages. However, it sharply increased to become the fifth and seventh leading cause for people aged 50 to 74 and 75 and older, respectively (3). Non-small-cell lung cancer (NSCLC) and small-cell lung cancer (SCLC) are two different types of lung cancer based on their histopathological characteristics (4). NSCLC is the most common type of lung cancer, accounting for 85% of cases. Adenocarcinoma (40%), squamous cell carcinoma (30%), and large-cell carcinoma (10%) are the three primary subtypes (5). Epidemiologic studies have shown that inflammation can induce tissue damage and help to develop lung cancer (6).

Cytokines are soluble chemical messengers that are critical in signaling immune responses. Th2 cytokines (IL-4, IL-5, and IL-10) steer the T-cell response away from a protective Th1 phenotype, while Th1 cytokines (IL-2, IFNγ, and TNFα) trigger cell-mediated responses. Malignant tumors have a skewed Th1/Th2 cytokine balance that encourages an immunosuppressive microenvironment (7). The primary regulators of the innate and adaptive immune systems, cytokines play a crucial role in regulating immunological responses to infections, autoimmune disorders, and cancer. Cytokines have a wide range of complex functions. In addition to providing protection, they can also contribute to developing cancer or autoimmune illnesses when overactive or severely deficient (8). Cytokines control the inflammatory response, which can have endocrine, paracrine, or autocrine actions. Besides producing inflammatory cytokines, human lung cancer cells can also do so (9). The different triggers that might result in the release of cytokines, which regulate numerous aspects of human physiology and disease locally, include interferons (IFNs), interleukins, colony-stimulating factors, chemokines, growth factors, and tumor necrosis factors (10). Recombinant cytokines have been used to treat cancer for a very long time because they are essential in developing, spreading, and eliminating cancer. However, the limited value of cytokines as therapeutics is due to their poor drug-like properties, complex biology, cytokine pleiotropy, severe dose-limiting toxicities, and drug-like characteristics of cytokines (11). Cytokines, however, offer the potential to enhance immunotherapy techniques for cancer treatment as they play a crucial role in innate and adaptive anti-tumor immunity. The classification of cytokines as inflammatory (released by Th-1 T helper cells) or anti-inflammatory (released by Th-2 T helper cells) is a frequent practice (12). The pathogenesis of cancer, particularly lung cancer, is thought to be significantly influenced by specific inflammatory cytokines, according to numerous research published to date (13).

According to many preclinical and clinical studies, immune cells that infiltrate tumors or tumor cells themselves may release cytokines and chemokines that alter the tumor microenvironment and encourage angiogenesis, growth, invasion, and metastasis. Additionally, cytokines work as a pro-tumor factor or a tumor cell growth inhibitor, respectively (14). According to reports, immunotherapy patients with metastatic melanoma with cytokine profiles evaluated throughout time had shown a correlation between the progression of irAEs and severe irAEs. In patients with advanced NSCLC receiving immunotherapy, recent studies have suggested that elevated levels of IL-1β and IFN-γ during treatment may indicate efficacy. In contrast, high levels of IL-6 during treatment may show less favorable results (15, 16).

Due to improvements in diagnostic and treatment methods and the fact that lung cancer is typically identified at a mature phase, patients’ prognoses are still direr for those with lung cancer than those with other types of cancer, with a 5-year comparative mortality risk of less than 20%. (17). The pillars of cancer treatment for a very long period included surgery, chemotherapy, and radiation therapy. The fourth pillar of the cancer care approach, immunotherapy, was recently added, giving patients their first opportunity to be treated for a possible treatment (18). Furthermore, in 2015, the Phase II Checkmate 063 study was the first significant study of immunotherapy showing activity in NSCLC (19). Immunotherapy is a cancer treatment employing the patient’s immune system. Immunotherapy can alter the immune system’s functioning to help it recognize and combat cancer cells (20). Chimeric antigen receptor (CAR) T-cell therapy, checkpoint inhibitors, cytokines, immunomodulators, and cancer vaccines are a few of the vital immunotherapy treatments for cancer currently being researched (21).

Over the past 40 years, much research has been done on cytokines and cytokine receptors as tumor targets and treatments. More excellent knowledge of the cancer ecosystem and the advancement of more efficient immunotherapies have led to the development of novel methods for employing cytokine channels in cancer therapy (22). Among these methods is the use of cytokine-based medicines to intensify other medications’ immune-related side effects and focus on tumors in their early stages (15). Elevated amounts of cytokines can be found in those with immune reactions to inflammation and cancer. Smoking is the primary risk factor for lung cancer, and smoke irritants and chemical irritants can change lung cancer cytokine levels by triggering an immune response (23).

Lung cancer still has an extremely poor prognosis despite all the advancements in treatment. This dismal outlook highlights the urgent requirement for the creation of new methods to prevent and cure this fatal illness successfully (24). The prognosis is the doctor’s best guess regarding how cancer will progress and respond to treatment. Numerous factors affect the prognosis and likelihood of survival. Performance status, age, and gender are examples of patient-related prognostic factors. Tumor-related prognostic indicators include clinical stage and histological type (25). Prognostic markers identify patient subpopulations with markedly varied expected outcomes who may benefit from various treatment modalities. However, a prognostic factor cannot provide information on a specific therapy’s advantages. In contrast, individuals with many unfavorable prognostic variables may require more active treatment to achieve the same lifespan as individuals without these qualities. Patients with many favorable predictive characteristics may require less intensive treatment to attain a cure.

In the earlier research, only a few cytokines were examined concurrently in the same subjects. The lack of prior studies reporting on the connection between lung cancer survival and circulating cytokine concentrations represents another significant gap in the evidence (26). The development of novel therapeutic targets and biomarkers can predict how well immune-based therapy for lung cancer will work, and a better understanding of these things is urgently needed.

## 2 Serum cytokine levels as possible immunotherapeutic targets

Small substances called serum cytokines play a crucial role in regulating other immune system cells’ development and function. When released, they give the immune system the go-ahead to function. All blood cell proliferation and the growth of other cells that support the body’s immunological and inflammatory systems are impacted by cytokines (27). By transmitting signals that can cause abnormal cells to die and normal cells to live longer, they also boost the body’s natural ability to fight cancer.

A chemokine is a special type of cytokine. Immune cells can be directed toward a target by chemokines. Chemokines come in various forms, including interleukins, interferons, tumor necrosis, and growth factors (28). In response to bacterial products and early response inflammatory mediators, resident lung cells secrete chemokines, which are small (~8–10 kD) heparin-binding proteins. These proteins are then preserved by matrix heparin sulfate proteoglycans at the site of inflammation, forming a chemokine gradient toward the inflammatory focus. Chemokines have chemotactic and activating actions on leukocyte subsets and serve as an essential stimulus for driving leukocytes to sites of injury. Chemokines are divided into four subfamilies based on the location of cysteine residues: CXC (α), CC (β), C (γ), and CX3C (δ) (29). The CXC (α) cysteine motif consists of the first two NH_2_-terminal cysteines separated by one nonconserved aa residue. Each subfamily stimulates a particular cell type by binding to specific receptors on inflammatory cells (30, 31).

The lung cancer chemokine axis that has attracted the most focus to date is CXCL12/CXCR4, which has been associated with increased invasiveness and metastatic risk (32). DARC-expressing A549 cells in lung cancer were found to have significantly decreased cellularity, higher necrosis, lower microvessel density, and less metastasis (33). D6 may slow the growth of human NSCLC by trapping specific chemokines such as CCL2, CCL4, and CCL5 (34). Furthermore, a phase 1 clinical trial of immunotherapy discovered that administering DCs expressing the CCR7 receptor ligand CCL21 into lung cancers could enhance immunity and cause the tumor to shrink (35). It was discovered that the CCR5 ligand CCL4 increased the expression of the stromal-derived factor-erythroid differentiation regulator 1 (ERDR1), which might assist in the survival of cancer cells (36). As a result, the CCL4/CCR5 axis appears responsible for the progression and proliferation of lung tumors. Future studies on individuals with lung cancer should be undertaken to assess the results.

### 2.1 Interleukins

Important inflammatory cytokines include interleukins (ILs). In the human genome, more than 50 interleukins and associated proteins are encoded (37). The growth and occurrence of human malignant tumors are thought to be intimately tied to interleukins, which are also clinically used as diagnostic indicators for lung cancer (38). Previously, IL-1β (beta) and IL-1α (alpha) performed pro-tumorigenic functions in many malignancies and are double the primary serum mediators in the IL-1 family. Increased IL-1β promotes lung cancer spread by triggering angiogenesis, tumor growth, invasion, adhesion, cytokine production, and tumor epithelial-to-mesenchymal transition (39). In addition, the use of serum interleukin-1β (IL-1β), interleukin-6 (IL-6), and interleukin-8 (IL-8) in combination with carcinoembryonic antigen (CEA) as a biomarker panel for the detection and prediction of lung cancer metastasis are being studied (40). For example, several investigations have identified IL-1β as a prognostic factor in lung cancer, with elevated levels of IL-1β in blood or tumor tissue associated with poor existence (41).

In the tumor tissue and serum of NSCLC patients, IL-33 levels were noticeably elevated. The *in vitro* function investigation showed that IL-33 strongly encourages NSCLC cell proliferation, migration, and invasion. IL-33’s pro-tumor effect in NSCLC was further supported by *in vivo* research. Additionally, lung cancer has a bad prognosis when IL-33 is expressed at a lower level (42). Lung cancer was linked to elevated levels of the serum cytokines IL6 and IL8, and high levels of IL8 raised lung cancer risk. Research has been shown to elaborate on the potential effects of IL-37 on tumor growth, immune responses, and tumor angiogenesis. It has been discovered to play an anti-tumor function in various tumor types, including non-small cell lung cancer (43). The non-small cell lung cancer (NSCLC) patient’s potential IL-37 regulation mechanism is poorly understood (44). IL-38, a recently discovered anti-inflammatory factor in non-small cell lung cancer (NSCLC), makes up around 85% of all lung malignancies (45).

The FDA approved the use of high-dose interleukin-2 (IL-2) therapy in 1992 for metastatic renal cell carcinoma and in 1998 for the treatment of metastatic melanoma (46). A different meta-analysis demonstrates that IL-2 combination medication effectively cures non-small cell lung cancer (NSCLC), increases overall survival, and has few harmful side effects (47). In individuals with advanced NSCLC, IL-2 serum level determination by regression analysis was demonstrated to have independent predictive value; as a result, its potential application for outcome prediction is advocated (48).

Human interleukin-3 (IL-3) assesses the tolerance, hematologic effects, and safety in individuals with small-cell lung cancer (SCLC) before and following multi-agent antineoplastic therapy in a randomized, placebo-controlled, double-blind study (49). Human lung tumor cell lines express interleukin 4 (IL-4) receptors, and IL-4 can mediate mild to moderate antiproliferative action *in vitro* and *in vivo* in animal examples of human lung tumors (50). Ten blood cytokine levels, including; granulocyte-macrophage colony-stimulating factor, TNF (alpha), and IFN (gamma), IL1, IL6, IL12, IL4, IL8, IL10, IL5, were compared between 296 European-Americans and 170 African-Americans to determine their associations with lung cancer (51).

Bezel et al. measured 16 cytokine levels of tumor necrosis factor-alpha (TNF-α), GM-CSF, interferon-gamma (IFN-γ), interleukins (IL): IL-6, IL-7, IL-13, IL-17A, IL-23, IL-1b, IL-2, IL-8, IL-10, IL-12p70, IL-4, IL-5) and fractalkine, in bronchoalveolar lavage fluid (BALF) and serum of individuals with lung cancer compared to healthy persons (52). In preclinical investigations, GM-CSF, CCL21, interleukin-2 (IL-2), IL-21, IL-12, IL-15, IL-18, and type 1 interferon have been found to have anticancer action and encourage the development of CD4 T cells into Th1 cells that can secrete anti-tumor cytokines, such as IFN-γ and IL-2 (53).

Our findings may offer new support for preventing and managing lung tumor-associated IPF (idiopathic pulmonary fibrosis), which Zhang et al. first discovered to have higher levels of IL-22, IL-23, and IL-17 in the serum of patients with IPF (54). According to several recent studies, interleukin-33 has been linked to the advancement of lung cancer and can have opposing effects on the disease depending on the circumstances (3). Initially produced by regulatory T cells, IL-35 is a component of the interleukin-12 cytokines family. Significant evidence suggests that elevated blood IL-35 expression is linked to non-small cell pulmonary cancer in individuals with stage IV NSCLC (55).

According to Zhao and their colleagues, individuals with pulmonary cancer who experienced immune-related adverse events (AE) following immunotherapy had greater pre-treatment levels of IFN-α, IL-2, and IL-17 than those who experienced nonimmune-related adverse events (p=0.002, p=0.01, and p=0.02, respectively) (NAE). Before the second cycle of medication, individuals with AE compared to those with NAE had significantly higher variations in IFN-α (p=0.003), IFN-γ (p=0.012), and TNF-α (p=0.049), IL-2 (p=0.04), and IL-5 (p=0.007) levels (30) (See **Table 1**).

**Table 1:** Serum interleukin families’ importance and function in lung cancer.

Interleukin		Function in lungs cancer	Potential therapeutic strategy	References
*IL-1 superfamily: IL-1 subfamily*				
IL-1α	Enhance squamous carcinoma-associated fibroblast proliferation		Not explored	(56)
IL-1β	Facilitates lung cancer metastasis, cytokine production, angiogenesis		Therapeutic neutralization to manage CRS in ACT, cancer prevention and treatment (CANTOS)	(44)
IL-33	promotes the proliferation, migration, and invasion of the NSCLC cells		Representing an effective and promising strategy for NSCLC treatment	(49)
*IL-1 superfamily: IL-18 subfamily*				
IL-18	Tissue remodeling and homeostasis, cell proliferation, and angiogenesis		Preclinical engineered rIL-18 or in combination with ACT, hampered by IL-18BP	(57)
IL-37	promoting lung cancer cell proliferation		Not explored	(33)
*IL-1 superfamily: IL-36 subfamily*				
IL-38	promoting lung cancer cell proliferation		significantly associated with reduction of CD8^+^ TILs and tumor progression	(58)
*IL-2 (common γ-chain) family*				
IL-2	Up-regulated in a variety of epithelial tumors, including SCLC		Treatment of SCLC has not been fully recognized.	(49)
IL-4	Lung cancer carcinogenesis and progression		is a new therapeutic target for the prevention and treatment of lung cancer.	(59)
IL-7	Lung cancer proliferation, prevention of apoptosis		Not explored	(60)
IL-9	Inhabit lung metastasis		a new approach for clinical therapy of lung cancer.	(61)
IL-15	Evasion of immune responses allows progression and dissemination of tumor.		Not explored	(62)
IL-21	Reduced the growth and invasion of NSCLC cells		novel molecular target for NSCLC diagnosis and therapy	(63)
*IL-3 family*				
IL-3	Hematopoietic factor promotes hematological malignancies		Fused toxins to target CD123-bearing cells	(64)
IL-5	Growth and metastasis of lung cancer		therapeutic benefits to prevent or treat lung metastasis.	(65)
*IL-6 family*				
IL-6	Promote invasion and metastasis		Predicting therapeutic targets in NSCLC.	(66)
IL-11	Promotes NSCLC cell invasion and migration		Not explored	(67)
IL-31	Lung cancer tumorigenesis		Not explored	(68)
*IL-10 family*				
IL-10	Enhanced angiogenesis of NSCLC		Not explored	(69)
IL-19	Understudied, evidently protumoural		Not explored	(70)
IL-20	Decreased expression of IL-20Rα in lung tissues of non-small lung cancer		provide a possible target for the treatment of lung cancer	(71)

### 2.2 Interferons

When a pathological compromise occurs in our body, interferons, which are cytokines, are naturally created by our cells. These chemical messengers make the nearby normal cells resistant to the same type of infection (72). Beta IFN-β, generated by fibroblasts, macrophages, and epithelial cells; gamma IFN-y, produced by activated T-lymphocytes and natural killer lymphocytes; and alpha IFNα, produced by leukocytes, are the three forms of interferon (IFNβ) (73). Interferons are a good treatment option for cancer since they control angiogenesis, have immunomodulatory abilities, and so forth. Even in the advanced stages of both SCLC and NSCLC lung cancer, interferons have been proven beneficial (74).

An anti-HER2 monoclonal antibody has been shown to target IFN-β and increase the immunocytokine’s potency compared to either agent alone in a previous investigation. These new results imply that trastuzumab-IFN-mutein warrants clinical study as a new contender for anticancer treatments (75). IFNs may have biological effects on small cell lung cancer, according to preclinical and clinical research (SCLC). Non-small cell lung cancer studies have indicated that IFN-alpha is ineffective (NSCLC). Nonetheless, the potential specific potentiation of cisplatinum by IFN against human NSCLC xenografts is a fresh, encouraging discovery (76). These preliminary findings show that chemotherapy with IFN-alpha and cisplatin may be a beneficial alternative treatment for those with advanced NSCLC (77). The GO/G1 phase of the cell cycle looked to be where the cells accumulated due to IFNα, having an antiproliferative effect (78). IFN α & β (type I IFN) has been extensively tested in numerous clinical cases and experimental lung cancer models and is effective. However, other types and subtypes of interferons, such as IFN gamma, have also been tested in numerous lung cancer cases with varying degrees of success (79).

In a direct cytotoxic impact, IFNα and IFNy work together synergistically to promote the lysis of cancerous cells (80). All three types of interferon increase the expression of cell surface antigens, such as tumor necrosis factor (TNF) receptors and MHC antigens, making tumor cells easier for cytotoxic leukocytes or TNF, a cytokine with cytostatic solid and cytotoxic effects, to recognize (81). Like other cancers of neuroendocrine origin, SCLC cells exhibit low endogenous levels of class I antigen expression. They may therefore be able to avoid immune monitoring and spread more quickly in living things (82). IFNa and IFNy cause both SCLC and non-small cell lung cancer (NSCLC) cells to express HLA-A, B, C, and 2 microglobulin on their cell surfaces (83). IFNy can induce class II MHC antigens. Crawford has proven that the peripheral blood monocytes of patients with advanced NSCLC are activated by IFNy, as shown by a 50% or greater increase in the expression of the Fc receptor and 2 microglobulins by these cells (84).

### 2.3 Tumor necrosis factors

The multifunctional serum cytokine tumor necrosis factor (TNF) is crucial for various cellular processes, including cell proliferation, differentiation, survival, and death. Inflammatory cells release TNF, a cytokine that promotes inflammation and may contribute to cancer development (85). Nuclear factor b (NF-B) and c-Jun N-terminal kinase are (JNK). NF-κB is just two of the different signaling pathways that TNF uses to carry out its biological actions (JNK). While chronic JNK activation promotes cell death, NF-κB is an anti-apoptotic key cell survival signal (86). In determining cellular responses to TNF, NF-κB and JNK interact. TNF is duplicitous in cancer (87). TNF enhances tumor cell growth, invasion, proliferation, and tumor angiogenesis, as well as metastasis. Hence TNF may be an endogenous tumor promoter. TNF, however, might prevent cancer from developing (88). TNF can cause the death of cancer cells, making it a potential cancer treatment. However, much work is needed to lower its toxicity before TNF may be administered regularly (89).

Several tissues produce TNF, which is induced to express itself in response to inflammatory stimuli such as lipopolysaccharides (LPS). Notably, TNF is known to be released by cancerous cells in the tumor microenvironment (90). Toxicity and endotoxic shock have severely restricted the use of TNF for therapeutic purposes. Additionally, numerous investigations have revealed that TNF plays an oncogenic function in cancers linked to inflammation (91). TNF activates several inflammatory signaling networks by binding to its cognate receptors, TNF receptor 1 (TNFR1) or TNF receptor 2 (TNFR2) (92). TNFR1 is extensively distributed. However, TNFR2 is expressed mainly by immune and endothelial cells. Lung cancer has been found to have high levels of the cytokine and its receptors TNFR1 and TNFR2 (93).

Non-small cell lung cancer has a high expression of TNF and its receptors (NSCLC). The transcription factor NF-BNF-κB is significantly activated by TNF. According to studies, EGFR suppression causes TNF to be quickly increased in NSCLC, and this overexpression activates NF-NF-κB (94). The serum mediator of innate immunity responsible for causing hemorrhagic necrosis in tumors, TNF-alpha, was first discovered in the 1970s (95). TNFα levels were previously examined in 28 new patients with advanced-stage NSCLC before and after chemotherapy, as well as 15 healthy controls. The results showed that NSCLC patients had greater levels of TNF-α than controls (96). The first clinically usable TNF family-based signature for predicting prognosis and chemotherapy effectiveness for patients with SCLC was developed because little is known about TNF’s functions in small-cell lung cancer. The results presented here offer a novel approach to assessing the outlook of SCLC individuals and enhancing clinical care (97).

Treatment for 16 individuals with advanced non-small cell lung cancer included low-dose interleukin-2 and tumor necrosis factor. Enhanced lysis of autologous tumor *in vitro* was shown in four out of four patients throughout therapy, and all patients had increased lymphokine-activated killer and natural killer activity. We conclude that low-dose interleukin-2 and tumor necrosis factor-a immunotherapy can mediate tumor regression while posing manageable harm (98, 99). Such findings imply that tumor necrosis factors may impart pulmonary cancer cells’ resistance to future reactive oxygen species-based therapies. This resistance may result from these cells’ enhanced production of manganese superoxide dismutase. Clinical therapy failures could occur, mainly if a tumor necrosis factor is administered along with other medications (100).

### 2.4 Transforming growth factor-beta

There are three isoforms of transforming growth factor-beta (TGF-β). TGF-β is a versatile cytokine typically overexpressed in advanced cancers and associated with a poor prognosis. TGF-β’s function is context-dependent. TGF-β inhibits cellular proliferation, promotes cell apoptosis, and reduces inflammation, acting as a tumor suppressor for pre-malignant cells. For advanced malignancies, TGF-β encourages immune escape, treatment resistance, and distant metastasis. TGF-β may control the actions of a variety of immune cells, including regulating the development of regulatory T cells (Tregs), lowering the cytotoxicity of T cells and natural killer cells (NKs), and suppressing the antigen presentation of dendritic cells (DCs) (101). In addition, TGF-β limits immune cell infiltration by promoting the production of peritumoral collagen. Anti-PD-1/PD-L1 therapy had a minimal impact on TMEs with overactive TGF-β signaling. The *TGFβ1* gene expression is higher in the tumor tissues of non-responders following anti-PD-1/PD-L1 treatments. In line with this, blocking PD-1/PD-L1 and TGF-β simultaneously produces an anti-tumor effect (102). Recently, a bispecific antibody (YM101) that targets both TGF-β and PD-L1 has been developed for cancer treatment (103).

**Figure 1:**
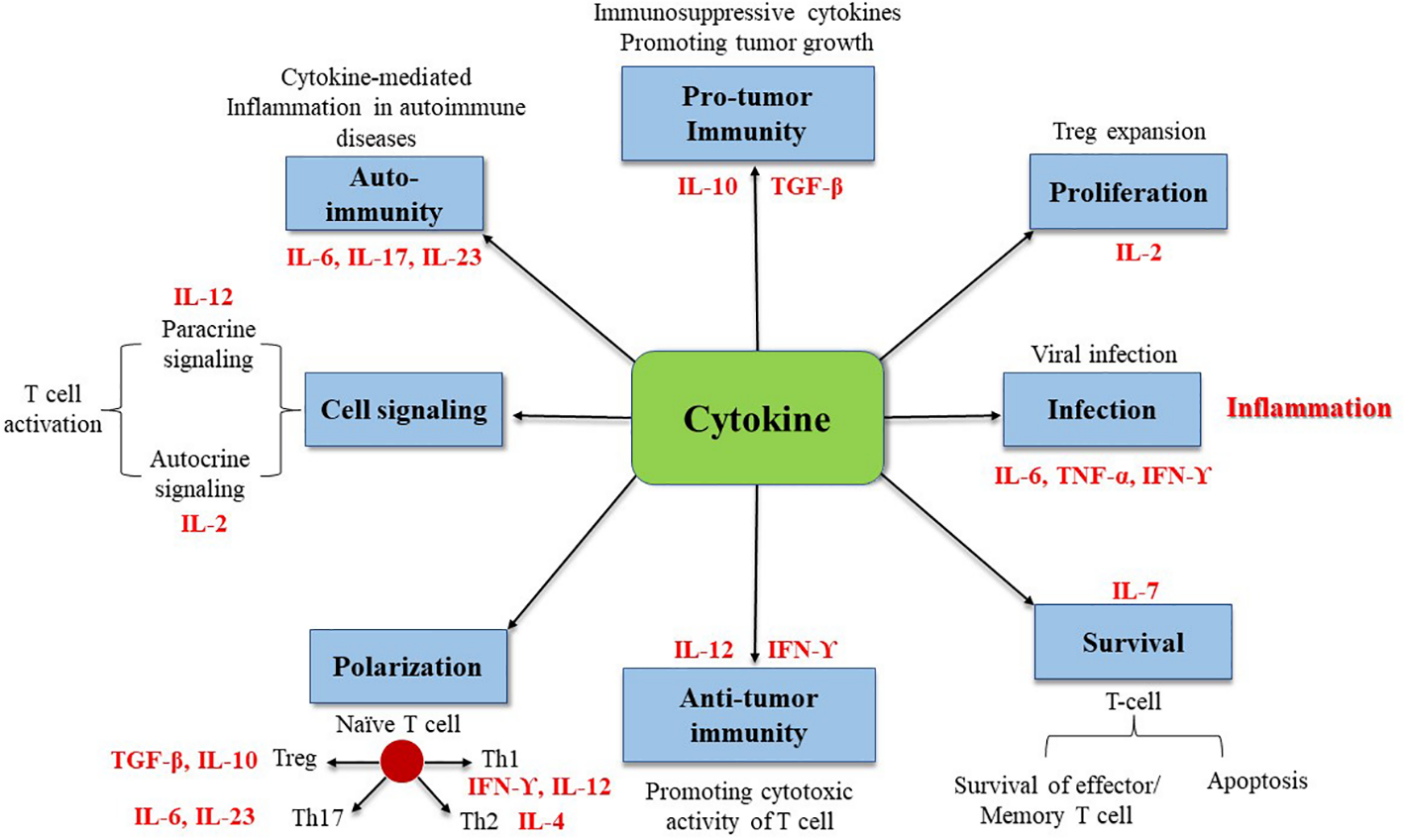
Illustration of cytokines involved in immune processes.

In summary, cytokines are the primary modulators of the innate and adaptive immune systems, primarily responsible for maintaining immunological homeostasis and regulating immune responses to infection, autoimmune diseases, and cancer. Cytokine roles are complicated and diverse. They can protect the body, but excessive activation or a severe lack can induce autoimmune disorders or promote cancer development (**Figure 1**).

## 3 Additional factors as prognostic indicators for lung cancer

Anorexia, performance status at the diagnosis, liver or skin metastases, and other conditions are additional indicators of a poor prognosis for NSCLC (104). According to molecular research, individuals with EGFR-activating mutations in adenocarcinoma had a better prognosis than patients without these mutations (105). Furthermore, the most significant prognostic factor for SCLC is the severity of the disease and the stage at presentation. The five-year mortality percentage for people with the restricted stage of cancer ranges from 10 to 13%, whereas the rate for those with the widespread phase of the infection is from 1 to 2%. (106)

It has been proposed that the following characteristics are linked to a better prognosis: Low microvessel count; low VEGF expression; absence of the KRAS mutation; no overexpression of c-erbB-2; No EGFR expression; TTF-1 positivity; high level of p16 expression; low-class III β-tubulin expression, low or no ERCC1 expression (advanced NSCLC treated with platinum-based chemotherapy); in individuals who had their tumors removed; low survivin expression. In a meta-analysis based on individual data, microvessel number was confirmed as a predictive factor for the prognostic value of angiogenesis, but only when it was estimated using the Chalkley procedure (107).

### 3.1 KLRK1

In a bioinformatics study, a Novel investigation found that KLRK1 is a prognostic and diagnostic biomarker for lung cancer (54). A homodimeric lectin-like receptor, KLRK1, encodes NKG2D. (108). KLRK1 is distinctive since it lacks inhibitory isoforms and functions as a cytotoxic and co-stimulatory molecule on T cells and NK cells. Two distinct KLRK1 ligands are found in the MIC and RAET1 gene families (109). KLRK1 is involved in regulating infection and tumor growth due to the frequent expression of its ligands on primary tumor cells (110). Phase I/II lung adenocarcinoma (P = 0.0094), older patients (P = 0.0072), male (P = 0.0033), and KLRK1 were all found to have substantial predictive significance. In phase I/II lung adenocarcinoma tumor individuals (P = 0.0025) and older patients (P = 0.012), KLRK1 was found to have substantial predictive significance (54).

### 3.2 ARL14

The expression of ARL14 in tumor tissues and standard samples was compared using immunohistochemistry (IHC). In NSCLC, we assessed ARL14’s predictive value. Higher ARL14 expression was linked to residual tumor in lung adenocarcinoma (P = 0.017), although it was related to age (P = 0.003) and N stage (P = 0.009) in lung squamous cell carcinoma. One hundred twenty patients with NSCLC provided similar outcomes. ARF7, FLJ22595, and ADP ribosylation factor-like GTPase 14 (ARL14) are protein-coding genes involved in GTP binding and signal transmission. ARL14 has recently been implicated in several biological activities, including vesicle-mediated and intracellular protein transport (111). Patients with bladder cancer with high levels of ARL14 methylation have a worse prognosis (112). ARF3, a homolog of ARL14, is said to express itself insufficiently in gastric cancer and may function as a possible biomarker for the prognosis of gastric cancer (113).

### 3.3 Gender as a prognostic factor

Women have a longer survival rate than men, according to numerous studies on gender as a prognostic factor in NSCLC. This may be related to biological variations between the sexes and/or confounding elements specific to women NSCLC individuals, such as smoking behaviors, histology, and young age (114). Individuals with small-cell lung cancer do not have gender-specific prognostic significance (SCLC). Eighty-eight women, or 14.9%, of the 591 patients with SCLC. Regarding minimal disease (48% vs. 37.8%) and no history of smoking (48.9% vs. 2.0%), women outnumbered males in likelihood. (52.3% vs. 62.8%) Men and women both had a progressive illness at the M stage. The complete cohort’s median survival times (MSTs) and 95% confidence intervals showed that women fared better than men in terms of survival (115). There were 144 NSCLC patients, all of whom had advanced disease. It was more common to see female patients. The most prevalent histologic type in both sexes (males, n = 54, 77.1%; females, n = 65, 87.8%) was adenocarcinoma (n = 119, 82.6%). (116).

### 3.4 TNM phase

According to the TNM classification, the clinical stage is the best indicator of prognosis in NSCLC (117). Distant metastases, tumor size, and nodal involvement are independently potent prognostic factors that are evaluated using the International Association for the Study of Lung Cancer (IASLC) international database of more than 80,000 lung cancer cases; the findings are thoroughly validated and frequently updated by the IASLC (118). However, within each stage group, there is much variation; some individuals advance their illness quickly, while others live for a very long time without experiencing a recurrence. Therefore, finding patient and tumor-related features is necessary to categorize patients according to their stage and individually tailor their treatments (119).

### 3.5 Age

The effects of age as a prognostic factor in NSCLC have been extensively explored. However, the outcomes are mixed. The medical features of lung tumors in individuals under the age of 45 are unusual and distinct from those in older people. Young individuals with advanced phase IIIB or IV (NSCLC) are being studied for their prognosis and outcomes. However, frequently, they are underrepresented in clinical trials, and older people (those over 65) with cancer have a high rate of cancer diagnoses (120). A randomized trial comparing the finest psychosocial support for individuals under 70 with adjuvant vinorelbine showed a substantial protective role and greater life satisfaction for the chemotherapy category (121).

### 3.6 Weight loss

The prognosis of many cancers, including NSCLC, is adversely affected by involuntary weight loss, a concerning indicator in oncology. Additionally, sarcopenia—the loss of skeletal muscle mass—is a potent predictor of poor prognosis distinct from weight loss (122). The proportion of body weight lost over a given period is a common way for writers to categorize weight reduction in their works. For patients with a BMI under 20, weight loss of more than 5% during the past six months (or more than 2%) qualifies as cancer cachexia (123). Patients who stabilize their weight after treatment have a better effect than those who continue to lose weight (124).

### 3.7 Performance status

Patients’ general health and everyday activities can be measured using their performance status. The ECOG score is more frequently used for performance status assessment in NSCLC because it is more widely utilized and easier to use than the KPS. It has also demonstrated higher predictive ability in patients with lung cancer (125). The inherent subjectivity of performance status and the weak-to-moderate connection in patient and doctor ratings are two issues that raise some doubts about its appropriateness. However, performance status is among the most well-researched prognostic markers, and its predictive value has been proven in several significant malignancies. The prevalence of low-Performance status in individuals with lung tumors compared to those with other cancers is not well understood, even though it is a common occurrence. Patients and clinicians estimated poor PS to be present in lung cancer patients at 34% and 48%, respectively (126).

### 3.8 Anemia

The typical definition of anemia is a reduction in the blood’s ability to carry oxygen due to a drop in hemoglobin (Hgb), a component of red blood cells. Hgb levels of less than 120 g/L for non-pregnant women and 130 g/L for men were used by a WHO study group in 1968 to identify anemia in adults (127). About 52% of tumor patients will become anemic during their illness, and 21% will need blood transfusions. Anemia is frequent in cancer patients (128). Anemia in cancer patients can be multifactorial and be brought on by cancer-related bleeding, cytokines produced by the tumor itself, or disruption of hematopoiesis brought on by metastatic bone marrow infiltration (129). Some studies suggest that pre-treatment anemia has a negative prognostic impact on NSCLC patients, but the function of anemia as a prognostic marker is debatable (130).

### 3.9 Leukocytosis

Patients with NSCLC typically experience leukocytosis, defined as an unusually high amount of WBCs in the peripheral blood. Infection, bone marrow metastases, and corticosteroids are some of the causes, either singularly or in combination (131). Nevertheless, other NSCLC patients exhibit leukocytosis for no apparent reason; tumor-related leukocytosis may be present (132). Interleukin-6, granulocyte-macrophage colony-stimulating factor (GM-CSF), and Granulocyte colony-stimulating factor (G-CSF) are three of the hematological cytokines that the tumor produces and secretes to create this paraneoplastic illness (133).

### 3.10 Thrombocytosis

It has long been understood that thrombocytosis, an unusually high amount of platelets in blood circulation, is associated with malignant illness (134). In the same way that leukocytosis can be a paraneoplastic condition, thrombocytosis can be brought on by an increase in the tumor’s cytokine production. Numerous studies have found that it is a poor predictor of survival for people with lung cancer (135). However, some studies do not find this correlation, such as a pooled study of the NCCT (North Central Cancer Treatment Group) findings, including information from more than 1000 individuals with advanced-stage NSCLC (136). These findings suggested that platelet counts and several clinical-pathologic traits could be helpful prognostic variables in patients with unresectable NSCLC. The patients were 347 consecutive phase IIIB or IV NSCLC individuals treated in the medical oncology department between 2005 and 2009. (137). According to meta-analyses, 27% of individuals with lung cancer had thrombocytosis overall. This was divided into 22% for adenocarcinoma, 28% for squamous cell carcinoma (SCC), 36% for large cell carcinoma (LCC), and 30% for SCLC (138). Thrombocytosis and a bad prognosis have been linked, but the exact mechanism is unclear (**Table 2**).

**Table 2:** The primary characteristics and clinical features of included studies.

Ref.	Year	Country	Sample size	Treatment	TNM stage	Threshold of SMI (cm2 /m2)	Tumor type	NOS
(139)	2015	United States	112	Non-surgery	IV	40	NSCLC	7
(140)	2016	Japan	90	Surgery	I	Male: 43.75; female: 41.10	NSCLC	7
(141)	2016	Norway	734	Non-surgery	III-IV	NR	NSCLC	7
(142)	2017	Japan	147	Surgery	I	Male: 43.75; female: 41.10	NSCLC	7
(143)	2017	Germany	200	Non-surgery	I-IV	NR	LC	7
(144)	2020	France	142	Non-surgery	NR	Male: 52.4; female: 38.5	NSCLC	6
(145)	2020	United Kingdom	643	Non-surgery	III-IV	Male: 43; female: 41	LC	6
(146)	2020	United Kingdom	119	Non-surgery	I-III	Male: 53; female: 41	NSCLC	6
(147)	2019	Israel	46	Non-surgery	IV	NR	LC	6
(148)	2021	Japan	60	Non-surgery	III	Male: 43; female: 24	NSCLC	7
(149)	2021	Republic of Korea	70	Non-surgery	IIIB-IV	Male: 46; female: 29	SCC	6
(67)	2021	China	639	Non-surgery	IIIB-IV	Male: 32.48; female: 27.82	NSCLC	7

*TNM, Tumor-node-metastasis; SMI, Skeletal muscle mass index; NOS, Newcastle-Ottawa Scale; NR, Not reported; NSCLC, Non-small cell lung cancer; LC, Lung cancer; SCC, Squamous cell cancer.

## 4 Conclusion and future perspectives

Finally, we determined that serum cytokine significantly impacts patients with lung cancer. In a disease where tumor biomarkers studies are challenging, baseline levels and treatment changes may serve as efficient predictive biomarkers of success from adding new drugs to treatment. This comprehensive evaluation of the literature revealed significant relationships between many cytokines and metabolic tumor burden, but the findings will need to be verified in a larger cohort.

## Author contributions

Study concept: YZ and LZ. Data collection: YZ, SJ, and KZ. Draft preparation: YZ, SJ, KZ, and LZ. Supervision: LZ. All authors read and agreed to the publication of this article.
