# Supplementary figures and images for: Serum cytokine levels and other associated factors as possible immunotherapeutic targets and prognostic indicators for lung cancer
Source: Front Oncol. 2023 Feb 16;13:1064616. doi: 10.3389/fonc.2023.1064616 (PMC9977806; doi:10.3389/fonc.2023.1064616)

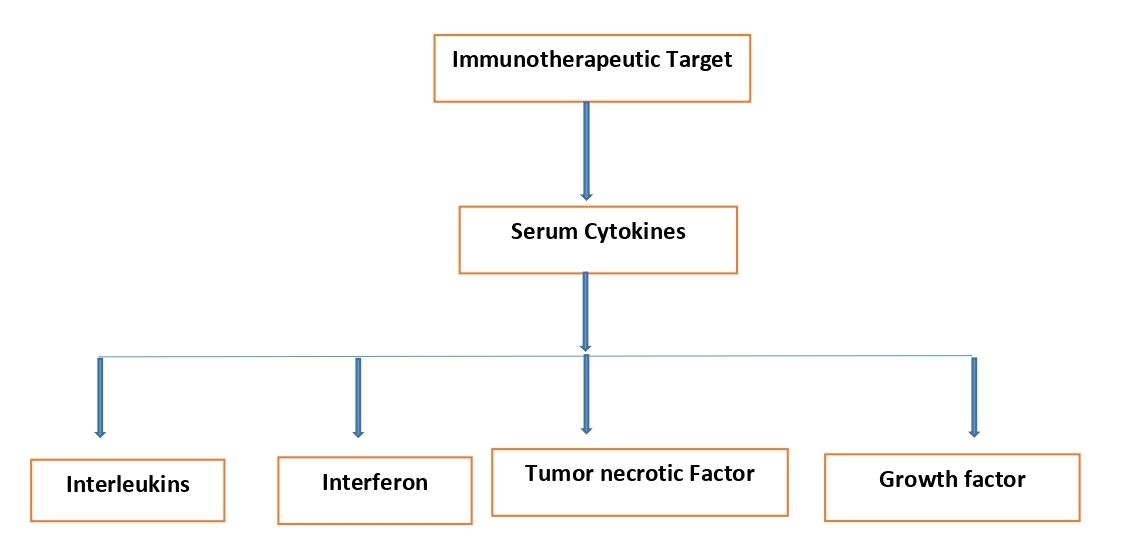

Supplement: Supplementary file 1 [file Image_1.jpeg]
